# Stereotactic body radiation therapy (SBRT) for prostate cancer: Improving treatment delivery efficiency and accuracy

**DOI:** 10.1016/j.tipsro.2024.100253

**Published:** 2024-05-06

**Authors:** Edoardo Mastella, Joel E. Epile, Eleonora De Guglielmo, Sara Fabbri, Francesca Calderoni, Luigi Manco, Klarisa E. Szilagyi, Antonio Malorgio, Alessandro Turra, Antonio Stefanelli

**Affiliations:** a Medical Physics Unit, University Hospital of Ferrara, via A. Moro 8, I-44124 Cona (Ferrara), Italy; b Master of Advanced Studies in Medical Physics, International Centre for Theoretical Physics (ICTP), Strada Costiera 11, I-34151 Trieste, Italy; c Medical Physics Unit, Azienda USL di Ferrara, via Cassoli 30, I-44121 Ferrara, Italy; d Specialization School of Medical Physics, University of Bologna, Viale Berti-Pichat 6/2, I-40127 Bologna, Italy; e Radiation Oncology Unit, University Hospital of Ferrara, via A. Moro 8, I-44124 Cona (Ferrara), Italy

**Keywords:** Volumetric modulated arc therapy (VMAT), Single arc, Ultra-hypofractionation, Flattening filter free (FFF) beam, Patient specific quality assurance (PSQA), Intrafraction motion mitigation

## Abstract

**Purpose:**

In stereotactic body radiation therapy (SBRT) for prostate cancer, intrafraction motion is an important source of treatment uncertainty as it could not be completely smoothed through fractionation. Herein, we compared different arrangements and beam qualities for extreme hypofractionated treatments to minimize beam delivery time and so intrafractional errors.

**Methods:**

A retrospective dataset of 11 patients was used. Three volumetric modulated arc therapy (VMAT) beam arrangements were compared for a prescription dose of 40 Gy/5 fractions: two full arcs, 6 MV flattening filter free (FFF); one full arc, 6 MV FFF; one full arc, 10 MV FFF. A plan quality index was defined to compare achievement of the planning goals. Plan complexity was evaluated with the modulation factor. Dose delivery accuracy and efficiency were measured with patient-specific quality assurance plans.

**Results:**

All treatment plans fulfilled all dose objectives. No statistical differences were found both in plan quality and complexity. Very accurate dose delivery was achieved with the three arrangements, with mean γ passing rates >96.5 % (2 %/2 mm criteria). Slightly but significantly higher γ passing rates were observed with single-arc 6 MV FFF. Contrariwise, statistically significant reductions of the delivery time were obtained with single-arc geometries: the average delivery times were 1.6 min (−46.1 %) and 1.3 min (−56.2 %) for 6 and 10 MV FFF respectively.

**Conclusions:**

The high-quality, very fast and accurate dose delivery of single-arc plans confirmed the suitability of this arrangement for prostate SBRT. In particular, the significant reduction of delivery time would improve treatment robustness against intrafraction prostate motion.

## Highlights

• Double- and single-arc VMAT plans were compared for linac-based prostate SBRT.
• Plans with high quality and low complexity were obtained with both arrangements.
• 2 % 2 mm mean gamma passing rates were above 96.5 %
• A significant reduction of delivery time (>56 %) was achieved with single arcs 10 MV FFF.
• The very fast delivery (∼1.3 min/8 Gy) improves robustness against intrafraction motion.

## Introduction

In the last years, the popularity of stereotactic body radiation therapy (SBRT) has increased significantly for the treatment of localized prostate cancer, based on the assumption of a lower α/β for the tumor than for normal tissues [1], [2]. Prostate SBRT is generally associated with low rates of side effects, with toxicities similar to those of conventional RT [3], [4]. However, unlike conventional or moderately hypofractionated regimens [5], SBRT offers a shorter treatment course that results in a significant improvement in patients’ quality of life. This reduced treatment time can be particularly beneficial for patients who may struggle with longer treatment courses or those seeking a quicker return to normal activities. Moreover, SBRT has the potential to significantly reduce waiting lists, allowing more patients to receive timely and effective care. This condensed approach could improve overall treatment efficiency and access within healthcare systems.

Extreme hypofractionated regimens are included in the last versions of the National Comprehensive Cancer Network (NCCN) guidelines for different risk groups [6]. Ultra-hypofractionation requires highly conformal dose distributions with very steep dose gradients and tight margins. On the other hand, intrafraction prostate motion is generally complex to take into account and is an important potential source of uncertainty especially when very few fractions are delivered. Indeed, with fewer fractions the impact of intrafraction errors is potentially greater as it could not be completely smoothed through fractionation. Furthermore, ultra-hypofractionated regimens and highly complex plans require longer delivery times, thus increasing the risk of intrafraction motion errors. Therefore, adequate motion mitigation strategies should be implemented. Different motion management devices have been introduced for guided treatments, such as ultrasound or electromagnetic tracking, but in clinical practice the usage of these devices requires time and human resources and may not be feasible for all patients. Therefore, as prostate displacements increase with time, a straightforward solution to reduce treatment uncertainties is to minimize delivery time [7].

Nowadays, prostate volumetric modulated arc therapy (VMAT)-SBRT treatments are commonly planned with multiple flattening filter free (FFF) arcs, generally two [8], [9], [10], [11], [12], [13], [14] but even up to four [15], thus requiring intrafraction motion monitoring.

The purpose of the present study was to implement and validate a VMAT-SBRT strategy to accurately treat low- and intermediate-risk prostate patients minimizing delivery uncertainties. Herein, single-arc (SA) geometries with FFF beams of different qualities were deeply evaluated in comparison with standard double-arc (DA) arrangement with the aim of reducing treatment delivery time and so uncertainties resulting from intrafraction prostate motion. To the best of our knowledge this is one of the first studies which provides robust data supporting this strategy for ultra-hypofractionated treatments in comparison with the commonly used DA arrangement. In particular, both planning evaluations and experimental results are presented to highlight the overall high plan quality of the proposed SA VMAT strategy for prostate SBRT on a traditional C-arm linac.

## Materials and methods

To enhance and quantify the quality of this work, the RATING guidelines [16] were used to prepare this treatment planning study.

### Patient cohort

A retrospective dataset of 11 low- and intermediate-risk prostate patients was used for the present analysis. To avoid bias in patient selection, we included patients consecutively treated with a prescription dose of 60 Gy/20 fractions [5]. For treatment simulation, patients were stabilized in supine position using the ProStep (IT-V, Innsbruck, AT) and the KneeFix (CIVCO Medical Solutions, Iowa, USA) systems attached to the couch. Computed tomography (CT) scans were acquired using a Philips Brilliance Big Bore CT scanner according to our clinical protocol and registered with multiparametric magnetic resonance imaging (mpMRI). Prior to treatment simulation and delivery, patients were asked to follow a preparation protocol similarly to Russo et al. [17] to limit prostate mobility and achieve anatomical reproducibility (full bladder, empty rectum). The clinical target volume (CTV) was defined as the whole prostate and the proximal 5–10 mm of seminal vesicles. The average CTV size was 43.9 cc (range 24.6–75.3 cc). An isotropic 6 mm expansion (except 3 mm posterior) was applied to define the planning target volume (PTV). The average PTV size was 99.1 cc (range 67.0–154.0 cc).

### Treatment planning

Treatments were planned with a VersaHD C-arm linac (Elekta AB, Stockholm, Sweden) equipped with a standard Agility multileaf collimator (MLC). Plans were optimized using Philips Pinnacle^3^ Evolution V16.4.3 treatment planning system (TPS). The prescription dose was set to 40 Gy in 5 fractions. The dose distributions were calculated with the collapsed cone convolution method and a 2 × 2 × 2 mm^3^ dose grid. Planning dose-volume constraints and priorities were defined for both target and organs at risk (OARs) as reported in Table 1. A commonly used VMAT arrangement, consisting of two 6 MV FFF full arcs, was compared to SA geometries with different beam qualities. The three investigated arrangements were the following:

• two full arcs; 6 MV FFF; gantry 178° to 182°, collimator 10°; gantry 182° to 178°, collimator 350°;
• one full arc; 6 MV FFF; gantry 178° to 182°, collimator 10°;
• one full arc; 10 MV FFF; gantry 178° to 182°, collimator 10°.

**Table 1:** Planning goals for prostate SBRT with a prescription dose of 40 Gy delivered in 5 fractions.

Structure of interest	Dose objective	Planning goal	Priority
CTV	D99% [Gy]	40	1

PTV	D99% [Gy]	38	1
	D50% [Gy]	40	1
	D0.03cc [Gy]	44	1

Rectum	D0.03cc [Gy]	42	1
	D5% [Gy]	40	2
	D10% [Gy]	36	2
	D20% [Gy]	32	2
	D50% [Gy]	20	2

Bladder	D0.03cc [Gy]	44	1
	D10% [Gy]	38	1
	D40% [Gy]	20	2

Penile bulb	D0.03cc [Gy]	40	3

A total of 33 VMAT plans were optimized starting from the same protocol, i.e. the same stored settings of objectives, weights and optimization parameters. The MLC motion was constrained to 0.5 cm/deg. A single high-experienced planner (≫100 SBRT patients) fine-tuned all plans to achieve the planning objectives and to improve dose distributions, without the time pressure of clinical planning. Plans were normalized to ensure at least 99% of the CTV received the prescription dose. To speed up the plan evaluation process, we created scorecards in the TPS to quickly visualise achievement of planning goals. All optimized plans were then discussed with an experienced radiation oncologist.

### Plan quality and complexity

The three arrangements were firstly evaluated in terms of dose volume histogram (DVH)-based metrics. In particular, the planning dose objectives of Table 1 were chosen as dose-summarizing parameters for plan comparison. Afterwards, a plan quality index (PQI) was calculated similarly to Jornet et al. [18] to summarize achievement of the planning goals of Table 1 among the techniques:

$$\mathrm{PQI}=\frac{{D99\%(CTV)}_{\mathrm{plan}}-{D99\%(CTV)}_{\mathrm{goal}}}{{D99\%(CTV)}_{\mathrm{goal}}}+\sum_{x=50\%,99\%}\frac{{\mathrm{Dx}(PTV)}_{\mathrm{plan}}-{\mathrm{Dx}(PTV)}_{\mathrm{goal}}}{{\mathrm{Dx}(PTV)}_{\mathrm{goal}}}+\frac{{D0.03cc(PTV)}_{\mathrm{goal}}-{D0.03cc(PTV)}_{\mathrm{plan}}}{{D0.03cc(PTV)}_{\mathrm{plan}}}+\sum_{i,y}\frac{{\mathrm{Dy}({\mathrm{OAR}}_{i})}_{\mathrm{goal}}-{\mathrm{Dy}({\mathrm{OAR}}_{i})}_{\mathrm{plan}}}{{\mathrm{Dy}({\mathrm{OAR}}_{i})}_{\mathrm{plan}}}$$

where *Dy* is the dose received by the *y%* of the *i*-th OAR. Higher PQI value corresponds to a better plan quality, i.e. a plan with more homogenous target dose distribution and/or lower dose to the OARs.

The modulation factor (MU/Gy) [19], was chosen to compare the overall complexity of the plans.

Statistical significance of the dosimetric differences was evaluated with the non-parameter Wilcoxon signed-rank test, using JMP Pro-V15.1.0 software (SAS Institute Inc, Cary, NC). A significance level of 5 % was chosen (p-value < 0.05). In the *post-hoc* analysis, the significance level of the dose-summarizing parameters was corrected using the Bonferroni method based on the number of multiple tests conducted.

### Dose delivery accuracy and efficiency

The planned dose distributions were delivered to the Octavius 4D phantom coupled with the 1500 Detector (PTW, Freiburg, Germany). The delivered and recalculated 3D dose distributions were compared with the γ analysis method [20] using the PTW VeriSoft V7.2 software. The γ passing rates were evaluated using 2 %/2 mm and 10 % dose threshold acceptance criteria with global normalization in absolute dose, following the American Association of Physicists in Medicine (AAPM) Task Group No. 218 guidelines [21].

Treatment efficiency was compared in terms of beam-on time (BOT) recorded during the delivery of the patient-specific quality assurance (PSQA) plan. For DA geometry, the BOT was recorded from the beginning of the first arc to the end of the second arc. The maximal achievable dose rate were 1400 MU/min and 2400 MU/min for 6 MV FFF and 10 MV FFF beams, respectively.

As above, a significance level of 5 % was chosen for statistical analysis.

## Results

### Plan quality and complexity

All treatment plans fulfilled all target coverage objectives and dose constraints to the OARs, without any protocol deviation. The optimization process of the three arrangements took approximately one working day per patient, with comparable planning times for the three groups. Table 2 summarizes the average values of the DVH metrics, the PQIs and the MU factors achieved with the different configurations. The differences between double and single arcs are shown in Supplementary Table S1 together with the corresponding p-values. On average, the dose to the OARs was drastically below the planning goals, ranging from −2.4 % (rectum D0.03 cc, SA 10 MV FFF) to −74.0 % (bladder D40 %, SA 6 MV FFF). In addition, more homogenous target coverages were achieved, with average PTV D99% and D0.03 cc values respectively above and below the planning objectives. The average PTV D99 % (D0.03cc) were 97.9 % (103.5 %), 97.1 % (104.4 %) and 97.1 % (104.3 %) of the prescribed dose respectively for DA 6 MV FFF, SA 6 MV FFF and SA 10 MV FFF. Although significant, very slight differences (∼0.8 %) were found in target coverage between double- and single-arc arrangements. With SA 10 MV FFF, a significant dose increment (1.1 %) was found in the bladder D0.03cc. When comparing 6 MV FFF and 10 MV FFF SA plans, no statistically significant differences were found in any of the analyzed parameters. The overall plan quality was comparable between all the three arrangements.

**Table 2:** Comparison of the metrics chosen for treatment plan evaluation. The mean values (±1 standard deviation) of the dose objectives, the plan quality indexes (PQIs) and the modulation (MU) factors are reported for the three arrangements. Statistically significant differences between double- and single-arc arrangements are highlighted in bold.

Comparison	Metric	Double-arc 6 MV FFF	Single-arc 6 MV FFF	Single-arc 10 MV FFF
CTV	D99% [Gy]	40.1±0.0	40.1±0.1	40.1±0.1

PTV	D99% [Gy]	39.2±0.2	**38.8±0.3**	38.8±0.4
	D50% [Gy]	40.4±0.1	40.5±0.1	**40.6±0.1**
	D0.03cc [Gy]	41.4±0.4	**41.7±0.3**	41.7±0.7

Rectum	D0.03cc [Gy]	40.8±0.4	40.9±0.4	41.0±0.4
	D5% [Gy]	38.2±1.3	38.1±2.1	38.0±1.3
	D10% [Gy]	33.3±3.1	33.8±3.3	33.6±2.8
	D20% [Gy]	24.4±2.8	24.8±2.8	24.4±2.7
	D50% [Gy]	13.6±2.5	13.0±2.8	12.8±2.3

Bladder	D0.03cc [Gy]	41.0±0.3	41.3±0.5	**41.5±0.4**
	D10% [Gy]	27.7±7.2	27.8±7.0	28.1±6.6
	D40% [Gy]	5.5±5.1	5.2±4.5	5.8±5.4

Penile bulb	D0.03cc [Gy]	28.1±8.6	29.0±8.3	28.7±8.4

Plan quality	PQI	8.6±5.0	8.6±5.0	8.5±5.3

Plan complexity	MU factor [MU/Gy]	197.5±21.4	191.9±16.9	195.5±19.1

*Note:* p-values are statistically significant after Bonferroni correction: p < 0.013 for CTV + PTV, p < 0.01 for rectum, p < 0.017 for bladder, p < 0.05 for penile bulb, PQI and MU factor.

**Fig. 1:**
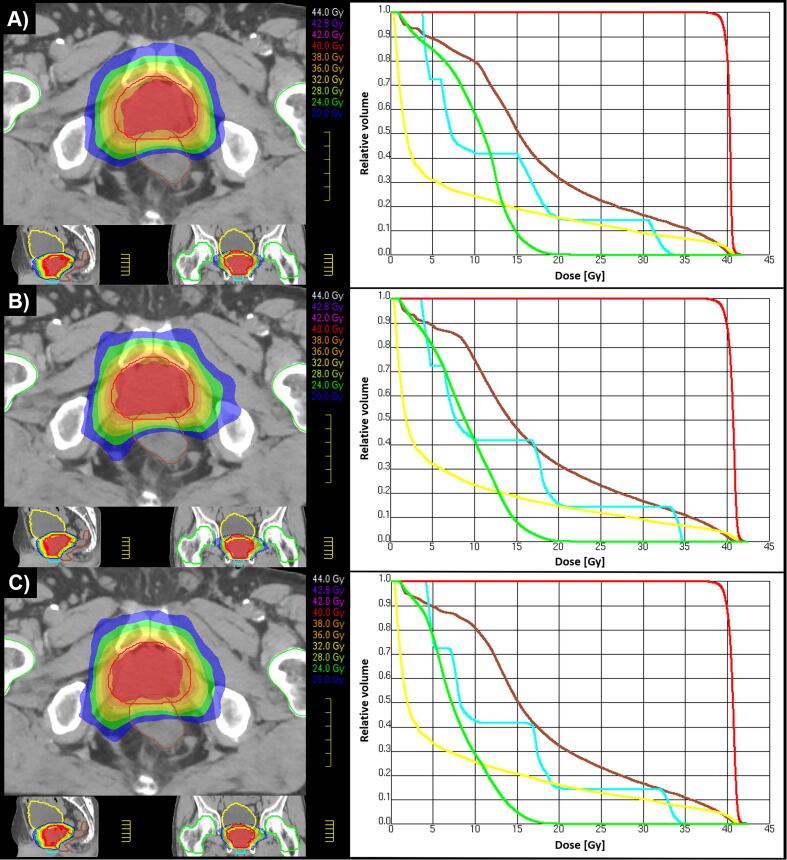
Dose distributions (on the left) and dose volume histograms (on the right) obtained using different arrangements for patient P11: A) two full arcs, 6 MV FFF, PQI = 7.9; B) one full arc, 6 MV FFF, PQI = 7.3; C) one full arc, 10 MV FFF, PQI = 6.6. The PTV is delineated in red, the rectum in brown, the bladder in yellow, the penile bulb in light blue and the femoral heads in green.

Fig. 1 shows examples of SBRT dose distributions and corresponding DVHs obtained with the three arrangements for a representative patient (P11, PQIs closer to the mean values).

Although not significant, slightly decreases of the MUs were observed with SA arrangements (−2.9 % and −1.0 % for 6 MV FFF and 10 MV FFF respectively).

### Dose delivery accuracy and efficiency

The BOTs and γ passing rates are summarized in Table 3, while the differences between double and single arcs are shown in Supplementary Table S2 together with the corresponding p-values. With DAs, the average BOT was almost 3 min, with a coefficient of variation (CV) of 3.5 %. Significant reductions of −46.1 % (6 MV FFF) and −56.2 % (10 MV FFF) of the BOTs was obtained with SAs. When comparing SA treatments, also the difference between the BOTs of two beam qualities was significant, with shorter times achieved with 10 MV FFF (mean = 1.3 min, CV = 3.6 %).

**Table 3:** Comparison of the dose delivery accuracy and efficiency. Mean values, standard deviations (SDs) and ranges of the beam-on time (BOT) and of the γ passing rate are reported for the three arrangements. Statistically significant differences (p < 0.05) between double- and single-arc arrangements are highlighted in bold.

Delivery parameter	Double-arc 6 MV FFF		Single-arc 6 MV FFF		Single-arc 10 MV FFF	
	Mean±SD	Range	Mean±SD	Range	Mean±SD	Range
BOT [min]	2.9±0.1	2.8÷3.1	**1.6±0.1**	**1.5÷1.8**	**1.3±0.0**	**1.2÷1.4**
γ passing rate [%]	96.7±0.9	95.1÷97.9	**97.3±0.8**	**95.5÷98.7**	96.6±1.4	94.5÷98.9

*Note:* For double-arc geometry, the BOT was recorded from the beginning of the first arc to the end of the second arc. γ analysis criteria: 2 %/2 mm, 10 % threshold, global normalization in absolute dose.

**Fig. 2:**
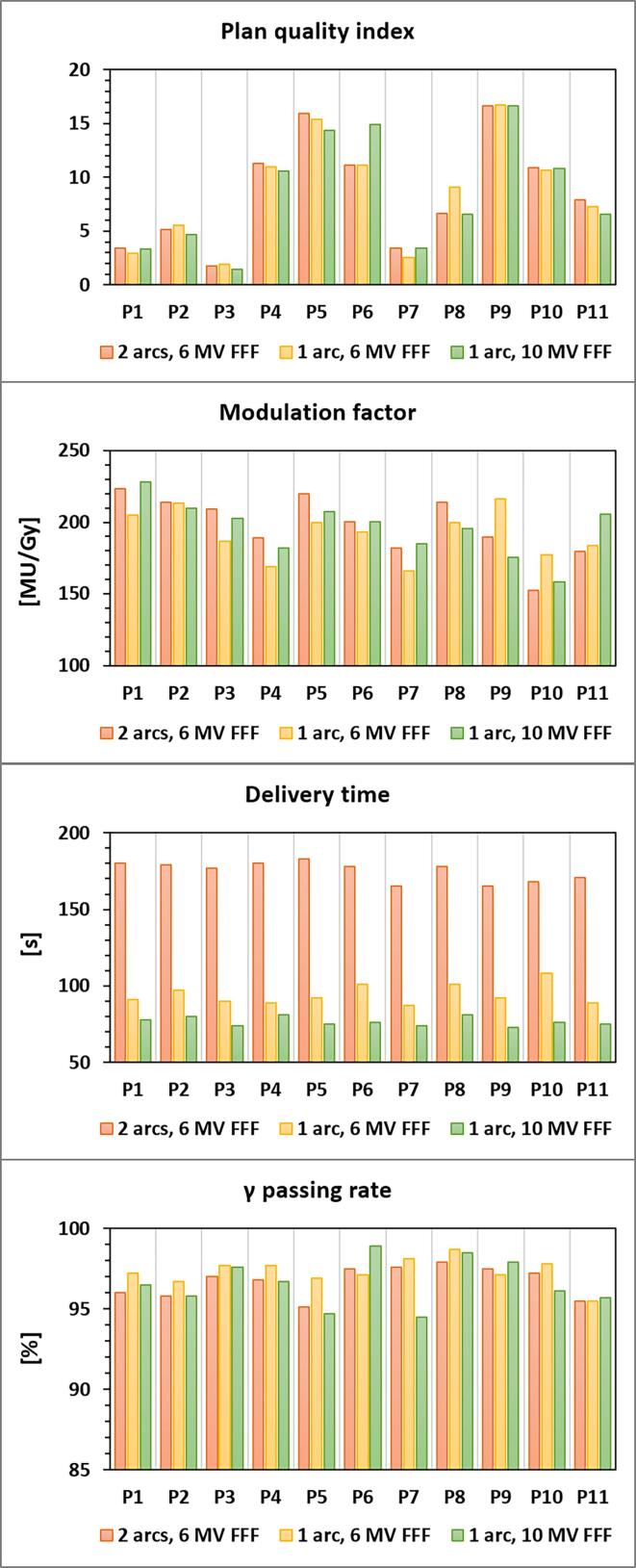
Plan quality index, modulation factor, beam-on time and γ passing rate reported for each patient P# and treatment plan arrangement.

All the mean γ passing rates were above the universal tolerance limits of 95 %. The largest variability was observed for 10 MV FFF, with the lowest (94.5 %, P7) and the highest (98.9%, P6) γ passing rates (CV = 1.4%). Slightly better results were observed with SA 6 MV FFF plans, with the highest mean passing rates (97.3 %) and the lowest CV (0.8%). When compared to DA (SA 10 MV FFF), the difference was (was not) statistically significant. All the PQIs, MU factors, BOTs and GPs are shown in Fig. 2.

## Discussion

In modern RT, a careful evaluation of treatment plans is a key step in the RT process [22]. In particular, in a SBRT program for prostate cancer, different highly qualified specialists should be involved in the standardization of the process due to complexity of the treatment workflow [23].

During radiotherapy, generally two types of prostate motion occur: a systematic drift of the prostatic gland largely in the posterior and inferior directions and a random motion, transient and often significant in extent mainly in the anterior and superior directions [24]. Therefore, PTV margins are expected to be a function of the beam-on time and any reduction in the overall treatment time, even small, is beneficial for patients, especially in prostate SBRT as higher fraction doses and highly complex plans require longer delivery times. Lovelock et al. [25] found that an additional 2-mm posterior margin should be added every 5 min after patient position verification. The authors also underlined that this is a minimum requirement not considering other factors which contributes to the need of even larger margins, such as deformation and rotation of the prostate. In addition, intrafraction motion of the seminal vesicles can be substantial and is largely uncorrelated with prostatic gland motion [7].

In this study, we have provided a deep overview of SA VMAT with the aim of achieving high-quality plans while significantly reducing treatment time and so intrafraction motion errors. For the first time, the correlations of plan quality, complexity, delivery accuracy and efficiency were investigated concurrently for different arrangements and beam qualities. According to the RATING guidelines [16], this study achieved a score of 90 % (see Supplementary materials).

There is still a debate on which is the optimal dose for low- and intermediate-risk prostate SBRT, with the most popular treatment schedules ranging from 35 Gy to 40 Gy delivered over 5 fractions [26], [27], [28], [29]. Herein, we investigated 8 Gy/fraction regimen as higher doses require longer delivery time. Anyway, these results can be extrapolated to treatment plans with lower fraction doses and similar complexities. Nonetheless, we are aware that a limitation of this study could be the small patient cohort, chosen for practical reason, and a greater number of patients would strengthen these results.

Target planning goals were defined similarly to our clinical protocol while OARs dose objectives and priorities were taken from the protocol of Lucchini et al. [11]. The objectives of the planning organ at risk volume for the urethra were not included in our analysis since they were guaranteed by the stricter objective for the maximum plan dose (D0.03 cc ≤ 44 Gy, i.e. 110 % of the prescribed dose) chosen to provide a significant dose-sparing of this structure. Similarly to [26], the strategy for femoral heads was to minimize the dose without compromising the other planning objectives. Differently from [11], conventional wider PTV margins were used as in our center real-time tracking is not implemented. To minimize potential planning bias and variability in plan quality, a single planner with high experience in SBRT optimized all treatment plans starting from the same protocol [16], [30]. To reduce plan complexity and so potential delivery inaccuracies, we constrained the MLC motion [31]. Highly homogenous dose distributions were achieved with maximum point doses always below 42.8 Gy (107 % of 40 Gy) and on average below 42 Gy (105%), further reducing the risk of treatment-related urinary toxicity without underdosing periurethral disease [32].

In treatment planning comparison, not only the achievement of the dose objectives but also the overall plan quality was reported. As underlined in [18], fulfilling all the dose constraints is not enough for plan comparison and a metric for plan quality evaluation is needed. The same overall plan quality was achieved with the three arrangements regardless the number of arcs and/or the beam quality. Focusing on single patients, very similar PQI values were obtained with both DA and SA. The indexes were mainly correlated to patient anatomy, i.e. the lowest and the highest PQIs were achieved for the same patient regardless the arrangement (see P3 and P9). Similarly to [18], we did not see any correlation between plan quality and complexity, e.g. patient P1 (P10) with the highest (lowest) MUs achieved PQI values below (above) the mean values. For prostate cancer treated with conventional fractionation, Guckenberger et al. [33] found a significant decrease in the MUs (∼20 %) for SA arrangements. Differently, we found comparable MUs for both different number of arcs and beam qualities. In general, a low degree of complexity was achieved with average modulation factors below 200 MU/Gy and CV around 10 %. Anyway, as more complex plans have larger uncertainties in dose calculation and treatment delivery [22], we delivered PSQA plans to evaluate if plan complexity correlated with delivery accuracy. Tight γ analysis criteria were chosen [21]. Very similar γ passing rates were observed with the three arrangements, with average values above the 95% universal tolerance limits [21]. Slightly but significantly higher γ passing rates were observed with SA 6 MV FFF, probably correlated with the lower MU factors. As expected, a significant improvement of treatment delivery efficiency was achieved with single arcs, with a significant reduction of BOT of more than 50% for 10 MV FFF. Moreover, the small variability of the delivery data indicated the reproducibility of our strategy for different patient anatomies and complexities.

Recently, Panizza et al. [12] evaluated the intrafraction prostate motion with a novel electromagnetic tracking device during the delivery of two 10 MV FFF arcs. They observed displacements below 2 mm in most of the cases within 10 min, with an increased probability of motion with time. With prescriptions of 40 Gy/5 fractions and 38 Gy/4 fractions, the mean delivery time was 3.5 min, which would have been reduced to 3.2 min without any intrafraction management.

Similar prostate displacements were found in other studies [34], [35], which highlighted that any reduction in overall treatment time could be beneficial for patients as reduces treatment uncertainties resulting from intrafractional motion. McNeice et al. [35] showed that the PTV margins used in our work (6/3 mm) adequately account for most of intrafraction motion.

Therefore, margins could be potentially decreased with our strategy due to the reduction in delivery time. However, this would require the analysis of the contribution to margins of other sources of errors, e.g. delineation errors [35], and will be investigated separately in a future study.

Faccenda et al. [13] compared the dosimetric impact of intrafraction prostate motion in linac-based gated and non-gated SBRT treatments using the same device, dose prescriptions and constraints described above [11], [12]. As expected, the authors found larger dose deviations in non-gated treatments due to intrafraction prostate motion. However, the effect of severe target degradations and bladder overdoses found in individual fractions was smoothed with fractionation in the cumulative dose. In our strategy, the high homogenous target coverage together with the low degree of plan complexity and the significant reduction of the BOT achieved using single arcs would further reduce deviations due to intrafraction motion, preventing potential overdosages in structures sensitive to radiation injury like the urethra.

The same authors, in a very recent retrospective study [36] re-planned DA treatments with single partial arcs (300° length) achieving a mean delivery time of 1.5 min, delivering 7.25 and 6.1 Gy/fraction and using 10 MV FFF. With increased planning and clinical experience, Panizza et al. [36] obtained higher quality SA plans increasing plan complexity while reducing the delivery time by 22 %.

In our work, despite a higher fraction dose (8 Gy), we achieved a lower mean BOT (1.3 min) using 10 MV FFF SA. Moreover, differently from [36] we obtained a larger reduction of delivery time (up to more than half) while maintaining the same plan quality and complexity. The reason of these discrepancies is probably related to the different degree of complexity in SA and DA plans between the two studies. In any case, the high plan quality achievable with SA VMAT and the strong agreement between the two studies in the delivery accuracy and efficiency further supports SA strategy for prostate SBRT.

Nowadays, specialized technologies and machines, e.g. the CyberKnife (CK) system (Accuray Inc., CA, USA) or MR-linac, allow real-time intrafraction motion management and on-line adaptive planning. Indeed, CK has been specifically developed to deliver SBRT treatments with very high precision and integrated real-time motion synchronization [37], while MR-linac enables advanced motion management and daily on-table adaptive planning to account for interfraction anatomic changes [38].

Recently, Ito et al. [14] compared the performances of the CK system with VMAT-FFF SBRT treatments and found an advantage of CK in terms of acute prostate symptoms. However, the authors concluded that SBRT is effective in treating prostate tumors without serious toxicity and regardless the treatment modality.

Several authors reported the potential of MR-linac for prostate SBRT [10], [39], [40], [41], [42]. In phase 3 MIRAGE randomized clinical trial, Kishan et al. [42] compared MRI-guided with CT-guided prostate SBRT and demonstrated that margin reduction allowed by MRI significantly reduced acute toxicities. On the other hand, the use of fiducial markers in CT guidance allows dosimetric accuracy comparable to MRI guidance [10]. In a very recent analysis, Giuliani et al. [43] concluded that the cost of managing relevant SBRT toxicities (low for both technologies) does not justify the difference in direct costs between adoption of MRI over CT guidance.

## Conclusions

High-quality plans with a low degree of complexity can be achieved with single-arc VMAT for prostate SBRT. In addition, the very fast, accurate and reproducible dose delivery of SA FFF treatments indicated the feasibility of the proposed strategy for low- and intermediate-risk patients. In particular, the significant reduction of the delivery time, especially when using 10 MV FFF, would improve treatment robustness against intrafraction motion errors and, as a consequence, patient safety.

## Funding statement

The authors received no specific funding for this work.

## Data availability statement for this work

All data generated and analyzed during this study are included in this published article (and its supplementary information files).

## CRediT authorship contribution statement

**Edoardo Mastella:** Conceptualization, Methodology, Validation, Writing – original draft, Visualization, Software, Investigation, Formal analysis, Data curation. **Joel E. Epile:** Software, Investigation, Formal analysis, Data curation. **Eleonora De Guglielmo:** Writing – review & editing. **Sara Fabbri:** Writing – review & editing. **Francesca Calderoni:** Writing – review & editing. **Luigi Manco:** Writing – review & editing. **Klarisa E. Szilagyi:** Writing – review & editing. **Antonio Malorgio:** Writing – review & editing. **Alessandro Turra:** Writing – review & editing. **Antonio Stefanelli:** Writing – review & editing. All authors contributed to the article and approved the submitted version.

## Declaration of competing interest

The authors declare that they have no known competing financial interests or personal relationships that could have appeared to influence the work reported in this paper.
