# Supplementary material for: Stereotactic body radiation therapy (SBRT) for prostate cancer: Improving treatment delivery efficiency and accuracy
Source: Tech Innov Patient Support Radiat Oncol. 2024 May 6;30:100253. doi: 10.1016/j.tipsro.2024.100253 (PMC11090872; doi:10.1016/j.tipsro.2024.100253)
Supplement: Supplementary Data 1 [file mmc1.docx]

**Supplementary Table 1.** Percentage differences of the mean values of the metrics chosen for treatment plan evaluation. The corresponding p-values are also reported. Statistically significant differences are highlighted in bold.

| **Comparison** | **Metric** | **Double-arc 6 MV FFF**  **vs**  **Single-arc 6 MV FFF** | **p-value** | **Double-arc 6 MV FFF**  **vs**  **Single-arc 10 MV FFF** | **p-value** |
| --- | --- | --- | --- | --- | --- |
| CTV | D99% | 0.2% | 0.024 | 0.2% | 0.019 |
| PTV | D99% | -0.8% | **0.010** | -0.8% | 0.019 |
|  | D50% | 0.3% | 0.067 | 0.4% | **<0.005** |
|  | D0.03cc | 0.8% | **0.010** | 0.8% | 0.019 |
| Rectum | D0.03cc | 0.3% | 0.18 | 0.5% | 0.18 |
|  | D5% | -0.4% | 0.70 | -0.6% | 0.32 |
|  | D10% | 1.4% | 0.12 | 0.7% | 0.64 |
|  | D20% | 1.5% | 0.37 | 0.0% | 0.58 |
|  | D50% | -4.3% | 0.24 | -5.7% | 0.042 |
| Bladder | D0.03cc | 0.7% | 0.067 | 1.1% | **<0.005** |
|  | D10% | 0.5% | 0.83 | 1.5% | 0.58 |
|  | D40% | -5.5% | 0.70 | 4.7% | 0.41 |
| Penile bulb | D0.03cc | 3.1% | 0.13 | 1.8% | 0.27 |
| Plan quality | PQI | 0.0% | 0.34 | -1.0% | 0.13 |
| Plan complexity | MU factor | -2.9% | 0.41 | -1.0% | 0.41 |

Note: The difference between the mean values is calculated as (1 arc – 2 arcs)/2 arcs x 100; p-values are statistically significant after Bonferroni correction: p < 0.013 for CTV + PTV, p < 0.01 for rectum, p < 0.017 for bladder, p < 0.05 for penile bulb, PQI and MU factor.

**Supplementary Table 2.** Percentage differences of the mean values of the dose delivery accuracy and efficiency. The corresponding p-values are also reported. Statistically significant differences are highlighted in bold.

| **Delivery parameter** | **Double-arc 6 MV FFF**  **vs**  **Single-arc 6 MV FFF** | **p-value** | **Double-arc 6 MV FFF**  **vs**  **Single-arc 10 MV FFF** | **p-value** |
| --- | --- | --- | --- | --- |
| BOT [min] | -46.1% | **<0.005** | -56.2% | **<0.005** |
| γ passing rate [%] | 0.6% | **0.014** | -0.1% | 0.61 |

Note: The difference between the mean values is calculated as (1 arc – 2 arcs)/2 arcs x 100

| **RATING score sheet** | | | Points | Applicable/ relevant | Answer yes |
| --- | --- | --- | --- | --- | --- |
| **Questions for the Introduction** | | |  |  |  |
|  | *The study aim formulated by research questions* | |  |  |  |
| 1 |  | Does the study have a concise and precise study aim, defined with a restricted number of interconnected questions? | 10 |  | 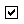 |
|  | *The motivation for the research questions* | |  |  |  |
| 2 |  | Has relevant up to date literature been included to support the need for the current study? | 5 |  | 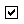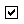   \|  \| \| --- \| |
| 3 |  | Does the study address an existing knowledge gap? | 10 |  |  |
| **Questions for Materials and Methods** | | |  |  |  |
| 4 |  | Is the global study design adequate for answering the posed research questions? | 10 |  | 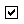 |
| 5 |  | Is the global study design described in sufficient detail for others to interpret and reproduce the results? | 5 |  | 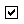 |
|  | *Patient cohort* | |  |  |  |
| 6 |  | Are the inclusion and exclusion criteria of the patient cohort described? | 1 | 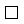 | 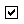 |
| 7 |  | Is the clinical patient information of the cohort presented, including disease type, site(s) and clinical staging? | 1 | 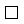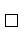   \|  \| \| --- \| | 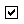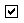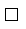 |
| 8 |  | Is the included number of patients stated, explained and justified? | 1 |  |  |
| 9 |  | Has there been consideration of the need for ethical and/or legal approval for the study and if needed, is there a statement about this? | 5 |  |  |
|  | *Imaging procedures* | |  |  |  |
| 10 |  | Have the scanning parameters been reported in sufficient detail (image modalities, equipment model, slice thickness, voxel size, patient position (e.g. head first, supine, etc.) etc.)? | 1 | 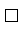 | 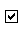 |
| 11 |  | Has the applied immobilisation equipment been described, (e.g. vendor and type, standard settings, etc.) where relevant? | 1 | 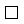 | 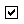 |
|  | *Treatment machine and settings* | |  |  |  |
| 12 |  | Have the treatment machine and relevant parameters been described with sufficient detail (model, beam energy, MLC, etc.)? | 1 | 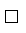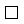 | 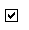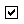 |
| 13 |  | Have the monitor unit reference conditions been defined, where relevant? | 1 |  |  |
|  | *Definition of targets and OARs* | |  |  |  |
| 14 |  | Has GTV definition been described in sufficient detail, with references if possible? | 1 | 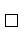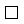 | 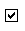 |
| 15 |  | Has CTV definition been described in sufficient detail, with references if possible? | 1 |  | 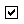 |
| 16 |  | Has the establishment of PTVs (or alternatively robustness settings) been described in sufficient detail? | 1 | 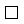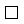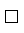 | 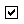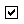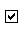 |
| 17 |  | Have PTV sizes in the patient cohort been described? | 1 |  |  |
| 18 |  | Have OAR definitions been described in sufficient detail, with references if possible? | 1 |  |  |
| 19 |  | Have PRV margins been described in sufficient detail, with references if available? | 1 | 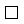 | 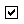 |
|  | *Treatment planning system and dose calculation* | |  |  |  |
| 20 |  | Have all applied dose calculation algorithms been described in sufficient detail? | 1 | 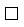 | 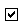 |
| 21 |  | For any commercial software used, have the manufacturer, algorithms and specific versions been stated? | 1 | 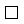 | 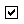 |
| 22 |  | Have all relevant user parameters and settings in the TPS been reported, e.g. beams, dose grid, control point spacing? | 1 | 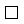 | 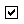 |
| 23 |  | Have all volumes been evaluated with the same software/methodology? | 1 | 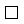 | 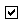 |
|  | *Planning aims and optimisation* | |  |  |  |
| 24 |  | Are clear planning aims defined, including imposed hard constraints and planning objectives (with or without soft constraints)? | 5 |  | 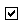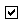 |
| 25 |  | Has the ranking of planning objectives (priorities) been described? | 5 |  |  |
| 26 |  | Is the dose prescription clearly defined? | 10 |  | 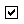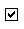 |
| 27 |  | Is there a narrative description of the applied optimisation process, including the handling of all objectives with their ranking? | 5 |  |  |
| 28 |  | If manual intervention during or after optimisation is allowed, has this been described? | 1 | 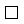 | 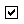 |
|  | *Bias mitigation* | |  |  |  |
| 29 |  | Have enough study details been provided such that bias issues could be noted? | 5 |  | 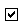 |
| 30 |  | Has bias been sufficiently mitigated to reliably answer the posed research question? | 10 |  | 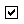 |
|  | *Plan acceptability – minor and major protocol deviations* | |  |  |  |
| 31 |  | Was the procedure for assessment of plan acceptability well-described? | 1 | 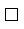 | 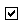 |
| 32 |  | Was the procedure for assessment of minor and major protocol deviations well described? | 1 | 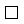 | 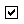 |
|  | *Plan (re-)normalisation for plan comparisons* | |  | 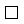   \|  \| \| --- \| | 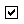   \|  \| \| --- \| |
| 33 |  | Has plan (re-)normalisation been described sufficiently? | 1 |  |  |
|  | *Dose-volume parameters for plan evaluation and comparison* | |  |  |  |
| 34 |  | Have sufficiently comprehensive dose-volume parameters been used for plan evaluations and comparisons? | 5 |  | 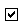 |
|  | *Population-mean DVHs* | |  |  |  |
| 35 |  | Has the algorithm for creating population-mean/median DVHs been reported? | 1 | 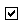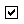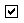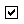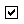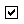   \|  \| \| --- \| | 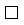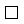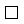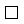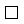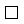   \|  \| \| --- \| |
| 36 |  | Have the definitions of confidence intervals been included? | 1 |  |  |
|  | *Plan evaluations by clinicians* | |  |  |  |
| 37 |  | Have clinicians scored plans to assess quality? | 1 |  |  |
| 38 |  | Were plan comparisons by clinicians blinded? | 1 |  |  |
|  | *Predicted tumour control probability and normal tissue complication probabilities for plan evaluation and comparison* | |  |  |  |
| 39 |  | Have any applied TCP models been described and referenced? | 1 |  |  |
| 40 |  | Have any applied NTCP models been described and referenced? | 1 |  |  |
|  | *Plan deliverability and complexity* | |  |  |  |
| 41 |  | Have methods used to assess plan deliverability and complexity been described in sufficient detail? | 1 | 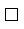 | 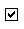 |
|  | *Composite plan quality metrics* | |  |  |  |
| 42 |  | Is there a sufficient basis (e.g. in the literature) for any selected composite plan quality metrics? | 1 | 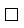 | 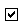 |
| 43 |  | Is there an adequate description of the calculation of the composite plan quality metrics? | 1 | 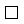 | 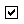 |
|  | *Planning and delivery times* | |  |  |  |
| 44 |  | Has measurement of planning times been described in sufficient detail? | 1 | 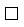 | 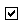 |
| 45 |  | Has the establishment of delivery times been described in sufficient detail? | 1 | 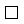 | 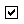 |
|  | *Statistical analysis* | |  |  |  |
| 46 |  | Have proper statistical methods been used and described in sufficient detail? | 5 |  | 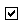 |
| 47 |  | In case of multiple testing for research questions, has this been handled appropriately? | 1 | 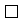 | 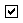 |
| **Questions for Results** | | |  |  |  |
| 48 |  | Does the provided data contribute to (at least partly) answering all aspects of the research questions, e.g. plan acceptability, dosimetric quality, deliverability and planning and delivery times? | 10 |  | 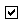 |
|  | *Dose distribution reporting* | |  |  |  |
| 49 |  | Are complete summaries of the dose distributions in the patient cohort provided (low doses, high doses, OARs, PTV, patient, etc.)? | 5 |  | 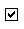 |
| 50 |  | Are tables and figures optimised to clearly present the results obtained? | 1 | 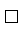 | 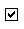 |
| 51 |  | Have the answers to the research questions been illustrated for an example patient by providing dose distributions, DVHs, etc.? | 1 | 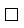   \|  \| \| --- \| | 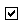   \|  \| \| --- \| |
|  | *Plan acceptability reporting – minor and major protocol deviations* | | |  |  |
| 52 |  | In case of treatment technique or planning technique comparisons, was plan acceptability reported separately for each technique? | 1 | 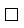 | 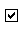 |
| 53 |  | Has plan acceptability been reported in sufficient detail: how many plans were acceptable, how many were not and for what reasons (e.g. violation of hard constraints, violation of soft constraints, other reasons)? | 1 | 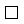 | 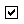 |
| 54 |  | Was there adequate reporting of minor and major protocol deviations? | 1 | 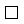 | 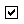 |
|  | *Deliverability and complexity reporting* | |  | 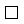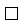   \|  \| \| --- \| | 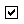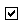   \|  \| \| --- \| |
| 55 |  | Has the deliverability of the plans been adequately reported? | 1 |  |  |
| 56 |  | Have plan deliverability and complexity been investigated in sufficient detail in relation to the posed research questions? | 1 |  |  |
|  | *Planning and delivery times reporting* | |  |  |  |
| 57 |  | Have planning and delivery times been adequately evaluated and reported? | 1 | 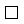 | 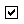 |
|  | *Patient-specific analyses reporting* | |  |  |  |
| 58 |  | Is there sufficient description of inter-patient variations in the results presented? | 1 | 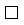 | 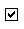 |
| 59 |  | Have outlier patients been reported and has any exclusion from population analyses been sufficiently motivated and explained? | 1 |  |  |
|  | *Statistical reporting* | |  | \|  \| \| --- \| |  |
| 60 |  | Are the p-values reported appropriately? | 1 |  | \|  \| \| --- \| |
| 61 |  | Are there confidence intervals for the appropriate parameters? | 1 |  |  |
| **Questions for discussions** | | |  |  |  |
| 62 |  | Is there an overall interpretation of the data presented in the Results section as to how the posed research questions are answered? | 10 |  |  |
|  | *Comparison with literature* | |  |  |  |
| 63 |  | Has the study been sufficiently discussed in the context of existing literature? | 5 |  |  |
|  | *Clinical and statistical significance* | |  | \|  \| \| --- \| | \|  \| \| --- \| |
| 64 |  | Does the discussion focus on statistically significant results? | 1 |  |  |
| 65 |  | Is the potential clinical significance of the results clearly discussed (assuming practical application would be feasible)? | 5 |  |  |
|  | *Clinical applicability of the study* | |  | \|  \| \| --- \| | \|  \| \| --- \| |
| 66 |  | Is future the clinical applicability sufficiently discussed? | 1 |  |  |
|  | *Study limitations* | |  |  |  |
| 67 |  | Has the impact of the study limitations on the provided answers to the research questions been sufficiently discussed? | 10 |  |  |
|  | *Future work* | |  | \|  \| \| --- \| | \|  \| \| --- \| |
| 68 |  | Has the potential future work arising from the study been discussed? | 1 |  |  |
| **Questions for conclusions** | | |  |  |  |
| 69 |  | Do the presented conclusions represent answers to the posed research questions? | 5 |  | \|  \| \| --- \| |
| 70 |  | Are the conclusions fully supported by the results? | 5 |  |  |
| 71 |  | Are the conclusions a fair summary of all results? | 5 |  |  |
| **Questions for supplementary** | | |  |  |  |
|  | *Supplementary materials* | |  |  |  |
| 72 |  | Is the information presented in the supplementary material of sufficient relevance? | 1 |  |  |
| 73 |  | Is the presentation of the included information of sufficient quality, including readability? | 1 |  |  |
| 74 |  | Has sufficient underlying data been made available or a willingness to share data been indicated, within local data sharing restrictions? | 5 |  |  |
| **RATING remarks** | | |  |  | \|  \| \| --- \| |
| 75 |  | Is the RATING score added to the manuscript? | 5 |  |  |
| 76 |  | Is the accompanying question table added to the cover letter or the supplementary material? | 1 |  |  |
|  |  |  |  |  |  |
|  |  | RATING score | 90% | | |
|  |  | **RATING fraction** | 155 | of | 173 |
